# Versatility of megakaryocytes in homeostasis and disease

**DOI:** 10.1097/BS9.0000000000000212

**Published:** 2024-11-28

**Authors:** Daosong Wang, Jiayi Xie, Meng Zhao

**Affiliations:** a RNA Biomedical Institute, Sun Yat-sen Memorial Hospital, Zhongshan School of Medicine, Sun Yat-sen University, Guangzhou, Guangdong, China; b Key Laboratory of Stem Cells and Tissue Engineering (Ministry of Education), Zhongshan School of Medicine, Sun Yat-sen University, Guangzhou, Guangdong, China; c Division of Blood and Marrow Transplantation, Department of Medicine, Stanford University, Stanford, CA

**Keywords:** Aging, Hematopoietic stem cell, Immune responses, Megakaryocyte, Niche, Platelet

## Abstract

Megakaryocytes (MKs) constitute a small portion of bone marrow cells and are primarily responsible for producing platelets, which are essential for hemostasis and wound healing. Recent studies have revealed that MKs and platelets perform diverse functions in various physiological and pathological contexts. This comprehensive review highlights the functional diversity of MKs beyond thrombopoiesis, including their roles in regulating hematopoietic stem cells, modulating immune responses, contributing to hematological malignancies, and influencing aging processes.

## 1. INTRODUCTION

Megakaryocytes (MKs) constitute approximately 0.05% of bone marrow cells and are distinguished by their large size (50–100 μm) and polyploid nuclei.^1,2^ Megakaryopoiesis and thrombopoiesis are essential and tightly regulated processes responsible for the efficient production of platelets. Beyond their role in platelet generation, MKs form part of the bone marrow niche, supporting the quiescence and regeneration of hematopoietic stem cells (HSCs).^3,4^ Furthermore, MKs participate in innate and adaptive immune responses.^5–9^ Traditionally, MKs are believed to arise from HSCs via the canonical hematopoietic hierarchy. However, recent evidence suggests that HSCs can directly differentiate into MK precursors, as demonstrated by in vivo barcoding, single-HSC differentiation, and transplantation experiments.^10–13^ Single-cell RNA sequencing (scRNA-seq) has revealed MK heterogeneity by identifying functionally distinct subpopulations involved in platelet production, HSC support, and immune responses in humans and mice.^5,6,14–17^ This review aims to provide a comprehensive overview of the functional diversity of MKs in physiological and pathological contexts.

## 2. MEGAKARYOPOIESIS AND THROMBOPOIESIS

### 2.1. Megakaryopoiesis

In the traditional hematopoietic hierarchy, MKs are believed to be generated through stepwise progression involving HSCs, multipotent progenitors, and lineage-restricted intermediates, including common myeloid progenitors, MK-erythrocyte progenitors (MEPs), and MK-committed progenitors (MKPs).^18,19^ However, this classical model of megakaryopoiesis has been revised in subsequent studies. For instance, the von Willebrand factor (vWF), a classical MK marker, identifies an HSC subset biased toward MK and platelet production upon transplantation.^20^ This finding introduces a novel approach for expanding MK-biased HSCs for platelet generation.^21^ Furthermore, single-HSC transplantation and differentiation experiments reveal that a distinct HSC subset can directly differentiate into the MK/platelet lineage, bypassing other blood cell lineages.^10–12^ This direct differentiation model was further supported by in vivo barcoding-based clonal analyses, which showed that the MK lineage is the predominant native fate of certain HSCs.^13^ These findings suggest a revised model in which HSCs can directly differentiate into MKs, in addition to the conventional stepwise progression model.

A recent scRNA-seq study has provided further insights, suggesting that different MK generation pathways may produce MKs with distinct functions. MKs generated via the direct differentiation pathway support the bone marrow niche, whereas those arising from the stepwise pathway participate in immune regulation. Platelet-producing MKs can be generated via both pathways.^17^ Additionally, the direct differentiation pathway from HSCs to MK progenitors is activated in pathological contexts, such as aging, leading to increased thrombocytosis and thrombosis.^22^ Furthermore, stem-like MKPs are activated for rapid MK and platelet production during inflammatory stress.^23^ MK-primed HSCs also expand in patients with the JAK2 V617F mutation in myeloproliferative neoplasms (MPNs).^24^ Moreover, HSC clones biased toward MK differentiation have been observed in patients with myelofibrosis^25^ and in mice infected with the influenza A virus.^25^ These studies suggest a role for MKs in aging and MPNs.

Thrombopoietin (TPO), originally referred to as MK-colony stimulating factor, binds to its receptor, TPO receptor (MPL), which is expressed in HSCs, MK progenitors, and mature MKs.^26,27^ The TPO-MPL axis is the key regulator of megakaryopoiesis, acting through the downstream JAK/STAT and ERK/AKT/CREB signaling pathways.^26,28^ In addition to its role in megakaryopoiesis, the TPO-MPL axis maintains HSC quiescence, genome integrity, and expansion.^29,30^ TPO is predominantly produced by hepatocytes in the liver, indicating a cross-organ regulatory mechanism for hematopoiesis and megakaryopoiesis.^31^ Furthermore, insulin-like growth factor I (IGF-1), which supports HSC maintenance and mitigates aging effects,^32^ promotes megakaryopoiesis via activation of the AKT signaling pathway.^33^ Stromal cell-derived factor-1 (SDF-1) and fibroblast growth factor (FGF) signaling also promote the maintenance and regeneration of HSCs.^34–37^ Moreover, IGF-1 facilitates the interaction between MK progenitors and the bone marrow vascular niche, thereby promoting megakaryopoiesis during homeostasis and after radiation-induced thrombocytopenia.^38,39^ These findings underscore the shared regulatory mechanisms governing HSCs and MKs.

Megakaryopoiesis is also influenced by various cell types in the bone marrow. Adipocytes regulate this process via CD36-mediated fatty acid transfer to MKs.^40^ Additionally, M2 macrophages and intravascular neutrophils support megakaryopoiesis by facilitating MK extensions, thereby modulating platelet production.^41,42^

### 2.2. Thrombopoiesis

MKs undergo endomitosis to become polyploid mature cells, which generate proplatelets, extrude their nuclei, and extend transendothelial proplatelets into the bone marrow sinusoids. Hemodynamic forces and environmental stiffness drive the elongation of these extensions, facilitating the platelet release into circulation. In humans, thrombopoiesis typically takes 5 days, whereas in mice, it occurs within 2 to 3 days.^2,43,44^

Thrombopoiesis heavily depends on cytoskeletal dynamics and polarization in MKs, which are processes tightly regulated by cellular metabolism. The mitochondrial fusion protein, mitofusin-2, plays a pivotal role in shaping mitochondrial morphology and controlling energy production in MKs, thereby influencing platelet activation and lifespan.^45^ Furthermore, lactate dehydrogenase A (LDHA) inhibits protein translation by physically interacting with eukaryotic elongation factor 2 in the cytoplasm, and the deletion of LDHA in MKs accelerates their maturation and platelet production.^46^ MEPs uptake kynurenine produced by colon, lung, or breast tumor cells via the SLC7A8 transporter, which activates the aryl hydrocarbon receptor-Runt-related transcription factor 1 axis to promote MEP differentiation into MKs.^47^ In multiple myeloma, MKs uptake environmental serine via SLC38A1, which downregulates supervillin through S-adenosyl-methionine-mediated H3K9 trimethylation, ultimately impairing megakaryopoiesis.^48^

Glycosylation plays a critical role in regulating megakaryopoiesis and platelet formation. Loss-of-function mutations in galactose metabolism and the protein glycosylation enzyme uridine diphosphate-galactose-4-epimerase have been identified in patients with lifelong severe thrombocytopenia. The impaired proplatelet formation observed in these patients is attributed to defective glycosylation of key proteins such as GPIbα and β1 integrin.^49^ Sphingosine 1-phosphate (S1P) supports the elongation of megakaryocytic proplatelet extensions into bone marrow sinusoids and their release into the bloodstream. The deletion of S1P receptor 1 results in severe thrombocytopenia.^50^ Phosphoinositide-dependent protein kinase 1 (PDK1), a key regulator of the phosphoinositide 3-kinase/Akt pathway, plays a crucial role in thrombin-induced platelet activation and arterial thrombosis formation.^51^ Loss of PDK1 in MKs disrupts actin cytoskeleton organization, reduces podosome formation, and impairs interactions between MKs and sinusoids, leading to MK hyperplasia and extramedullary thrombopoiesis.^52^

## 3. NICHE-SUPPORTING FUNCTION OF MKs FOR HSCs

MKs are in direct physical contact with HSCs in the bone marrow and play a crucial role in regulating HSC quiescence by secreting platelet factor 4 (PF4) and transforming growth factor-β (TGF-β).^3,4,53^ The MKs supporting HSCs are characterized by high ploidy and large cytoplasmic areas.^54^ These MKs preferentially interact with a vWF-expressing HSC subset.^55^ This HSC subset also expresses high levels of the non-receptor type protein tyrosine phosphatase SHP-1, a downstream effector of the TGF-β pathway.^56^ MK-derived TGF-β regulates not only HSC differentiation into erythroid lineages^57^ but also inhibits HSCs in acute myeloid leukemia.^58^ The production of TGF-β in MKs is regulated by phosphatidylinositol transfer proteins.^59^ MKs also produce TPO and potentially other niche factors controlled by the membrane protein C-type lectin-like receptor-2, which supports HSC function.^60^ Under stress conditions such as chemotherapy or radiation, MKs proliferate and secrete FGF 1 to promote HSC expansion and support their osteoblastic niche for regeneration.^36,61^ During embryonic development, MKs regulate HSCs in the aorta-gonad-mesonephros (AGM) region.^62^ Additionally, MKs produce IGF-1, which supports the maintenance and regeneration of the adult skeletal system.^63^

## 4. REGULATION OF IMMUNE RESPONSES BY MKs AND/OR PLATELETS

MKs and platelets actively participate in immune responses. MKs express a wide range of immune receptors, including IgG Fc receptors, toll-like receptors, interleukin (IL) receptors, and interferon receptors, allowing them to directly detect inflammation.^7^ In addition, mature MKs express major histocompatibility complex molecules, enabling them to activate CD8^+^ T and Th17 cells.^64,65^ Following inflammatory stress, the number of CD53-expressing MKs increases. These MKs have relatively low ploidy but show higher expression of immunological and inflammatory genes.^14^ In models of arthritis with Kit insufficiency, MKs produce IL-1 to promote inflammation.^66^ Lung MKs are hypothesized to present antigens and activate T cells based on their gene expression profiles.^67^ Human MKs and platelets express interferon-induced transmembrane protein 3 to combat dengue virus.^68^ MKs and platelets are also indicated in the immune response after COVID-19 infection.^69–71^ Moreover, human CD148- and CD48-expressing MKs show high immune receptor and mediator expression levels.^16^ MKs differentiated from human embryonic stem cells also strongly express immune response genes.^15^

MKs expressing high chemokine receptor levels migrate into the circulation and infiltrate the spleen, liver, and lungs upon bacterial infection. *Scl-CreERT*-based hematopoietic stem/progenitor cell lineage tracing experiments have shown that CXCR4^high^ MKs are produced via infection-induced emergency megakaryopoiesis.^5^ While normal HSC-to-MK development takes 11 to 12 days in humans and 4 days in mice, emergency megakaryopoiesis allows MK generation in less than a day in response to inflammatory stress.^72^ This suggests that CXCR4^high^ MKs are rapidly produced in the bone marrow to enhance host defenses in other tissues. These MKs secrete tumor necrosis factor (TNF-α) and IL-6, which stimulate bacterial phagocytosis by macrophages and neutrophils, and they also directly phagocytose bacteria, presenting antigens to activate T cells.^5^

During the onset of inflammation, platelets and neutrophils are recruited to the lungs. These cells together with regulatory T cells promote an anti-inflammatory macrophage phenotype, thereby reducing pulmonary inflammation.^73^ Platelets can also activate neutrophils, forming neutrophil extracellular traps in severe sepsis.^74^ This platelet-neutrophil cross-talk further contributes to the immune and complement responses during thrombotic vascular occlusion.^75^ However, the individual contributions of MKs and platelets to immune responses remain poorly understood because limited genetic models exist for distinguishing between the roles of MKs and platelets.

## 5. MKs IN MPNs

Myelofibrosis, a subtype of MPNs, is characterized by thrombosis, bone marrow fibrosis, myeloproliferation, extramedullary hematopoiesis, splenomegaly, and progression to leukemia.^76^ Multiple lines of evidence suggest that MKs play a central role in driving myelofibrosis. In mice, TPO overexpression leads to the development of myelofibrosis, which correlates with an increase in MK numbers.^77^ Similarly, GATA1 deficiency results in elevated immature MK levels and severe myelofibrosis.^78^ An increase in MK numbers in patients with myelofibrosis has been linked to higher IL-13 levels^79^ and widespread mutations in the calreticulin gene, which impair calcium release from the endoplasmic reticulum.^80^ However, the exact mechanism of increased MKs contributing to MPN pathogenesis remains unclear. Atypical MKs are believed to contribute to myelofibrosis through TGF-β release, a process that the JAK inhibitor ruxolitinib and aurora kinase a (AURKA) inhibitors can target.^81^ Mutations in MK progenitors may also lead to leukemic transformation. Activation of the BMP2/SMAD pathway in JAK2/p53-mutant MK-erythroid progenitors has been shown to promote MPN development.^82^ The transcriptional regulator PR/SET domain-containing 16 can also transform MK-erythroid progenitors into myeloid leukemia stem cells.^83^

## 6. MKs AND PLATELETS IN AGING

The risk of thrombosis increases with age owing to elevated platelet counts and heightened platelet reactivity, potentially driven by age-related inflammatory stressors such as activated TNF-α^84^ and mechanistic target of rapamycin complex 1 (mTORC1)^85^ signaling. PF4, produced by MKs, decreases in the plasma during aging in mice and humans. Systemic administration of PF4 can rejuvenate the aging immune system, mitigate neuroinflammation, and restore synaptic dynamics and cognitive functions in the aging hippocampus.^86–88^ MKs also influence the aging of neighboring HSCs in the bone marrow. With age, the direct differentiation pathway from HSCs to MKs becomes activated and decoupled from other hematopoietic lineages.^22^ Furthermore, aging is associated with increased noradrenergic innervation in the bone marrow, promoting megakaryopoiesis via a β2-adrenergic receptor (AR) and IL-6-dependent pathway. Treatment with a β3-AR agonist has been shown to restore the proximity of HSCs to MKs and reduce HSC aging.^89^ These findings suggest that aged MKs may play a role in systemic aging processes.

## 7. CONCLUDING REMARKS AND FUTURE PERSPECTIVES

**Figure 1.**
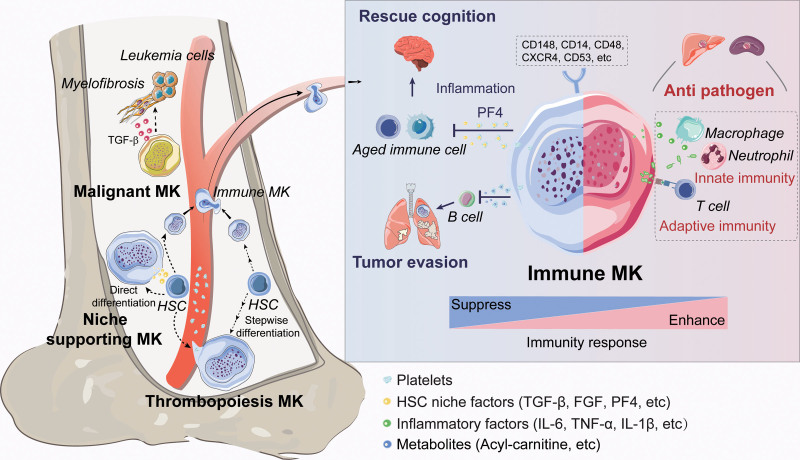
Versatility of MKs in homeostasis and disease. MKs play multifaceted roles in thrombopoiesis, supporting the hematopoietic stem cell niche and participating in immune responses. Abnormal MKs contribute to the development of myelofibrosis and myeloproliferative neoplasms within the bone marrow. In addition, immune MKs and/or platelets are implicated in various immune functions, including defense against pathogens, anti-inflammatory activities during aging, and the regulation of immune surveillance for tumors across multiple tissues. FGF = fibroblast growth factor, IL-6 = interleukin-6, IL-1β = interleukin-1β, MKs = megakaryocytes, PF4 = platelet factor 4, TGF-β = transforming growth factor-β, TNF-α = tumor necrosis factor.

This review highlights the remarkable versatility of MKs in physiological and pathological contexts (**Fig. 1**). However, a critical question remains: Do MKs perform these essential functions, or are they primarily mediated by their platelet derivatives? Under normal conditions, MKs are largely confined to the bone marrow and lungs, whereas platelets express a range of functional factors and circulate extensively throughout the body. This raises the possibility that platelets act as “large exosomes” and systematically influence tissue homeostasis, regeneration, and disease processes. Nonetheless, recent studies have suggested that certain MK subpopulations can migrate to various organs, contributing to local immune responses. To fully understand the roles of MKs and platelets, further investigations are necessary to distinguish their functions and elucidate their contributions to systemic and tissue-specific processes.

Recent studies have revealed that MKs from the embryonic AGM region and adult bone marrow share similar regulatory functions in HSCs. Additionally, lung-resident MKs exhibit transcriptional signatures closely resembling those of immune MKs in the bone marrow. These findings underscore the importance of further investigations to clarify how MK diversity is influenced by their source organs, developmental stages, ultrastructural properties, and molecular characteristics. A deeper understanding of MK heterogeneity will enhance efforts to generate functional MKs for clinical platelet production and pave the way for developing innovative MK- and platelet-based therapies aimed at tissue regeneration and treating various diseases.

## ACKNOWLEDGMENTS

This work was supported in part by the National Key Research and Development Program of China (2022YFA1104100), the National Natural Science Foundation of China (82325002).
